# Effect of Cassia Gum on the Gel Properties of Wheat Flour–Tapioca Starch-Based Batter and the Oil Absorption Capacity of Fried Small Yellow Croaker

**DOI:** 10.3390/gels11060469

**Published:** 2025-06-18

**Authors:** Guilian Ran, Qiang Zhang, Yiping Liao, Liufang Xu, Qiang Zou

**Affiliations:** 1 School of Food and Biological Engineering, Chengdu University, Chengdu 610106, China; 212023095135053@cdu.edu.cn (G.R.); zhangqiang@stu.cdu.edu.cn (Q.Z.); liaoyiping@cdu.edu.cn (Y.L.); xuliufang@cdu.edu.cn (L.X.); 2 Meat Processing Key Laboratory of Sichuan Province, School of Food and Biological Engineering, Chengdu University, Chengdu 610106, China

**Keywords:** cassia gum, batter property, fried food, oil penetration

## Abstract

High oil content in breaded fried small yellow croaker (BFYC) was reduced using composite batter gels consisting of tapioca starch, wheat flour, and different concentrations of cassia gum (CG; 0%, 0.2%, 0.4%, 0.6%, 0.8%, 1%). The effects of CG on the oil absorption capacity of BFYC and potential mechanisms were investigated. Dynamic rheological analysis revealed that CG addition could enhance the viscoelasticity of the batter by increasing its storage modulus and loss modulus. Furthermore, FTIR and X-ray diffraction results demonstrated that CG interacts with starch through noncovalent interactions, increasing the relative crystallinity from 9.29% to 16.49%, which promoted the formation of a gel layer. This structural improvement effectively inhibited oil absorption. Differential scanning calorimetry analysis showed that within the 0–0.8% CG range, the batter’s denaturation temperature increased from 78.23 °C to 82.08 °C with higher CG concentrations, indicating prolonged gelatinization and enhanced thermal stability that further reduced oil penetration. Low-field nuclear magnetic resonance analysis revealed that CG increased the proportion of tightly bound and weakly bound water in the batter, thereby improving water retention capacity and reducing moisture loss during frying. Microscopic structural observations and Sudan Red-staining tests confirmed that at 0.8% CG concentration, the crust exhibited the lowest porosity with approximately 40% reduction in surface fat content compared to the control group. In conclusion, CG addition significantly improves batter properties and reduces oil content in fried products, providing theoretical support for the development of low-fat fried foods.

## 1. Introduction

Traditional fried foods are prepared by coating ingredients with a batter typically composed of flour, starch, and protein, followed by high-temperature frying. During frying, the batter comes into contact with hot oil, accompanied by mass and heat transfer [1]. Under high temperatures, the batter undergoes a series of physical and chemical changes, such as gelatinization of starch, water evaporation, and protein denaturation, which impart a crispy texture and distinctive flavor to fried foods [2]. Battered and fried small yellow croaker (BFYC) is highly popular in Asian markets. However, it may contain excessive fat, and long-term consumption of high-fat fried foods can lead to obesity and chronic diseases, such as neurological disorders and cardiovascular diseases [3]. Consequently, strategies to reduce fat content while preserving the sensory characteristics of fried foods are strongly advocated.

**Figure 1:**
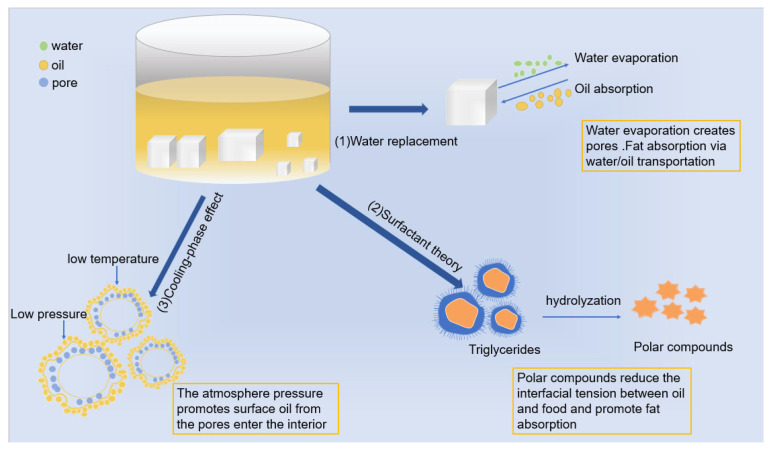
Schematic of fat absorption in fried and battered foods.

Oil absorption in fried, battered foods during frying is related to a variety of factors, such as food structure, type of edible oil, and frying conditions [4]. Qi et al. [5] investigated the effects of four types of frying oils on oil absorption in fried instant noodles. The results showed that oils containing 50% oleic acid (oleic sunflower oil/soybean oil/palm oil ratio of 24:25:1, *v*/*v*/*v*) were the most suitable. Zhai et al. [6] deep-fried hanging battered fish pieces at different temperatures (150–190 °C) and times (30–150 s). They found that fish pieces deep-fried at 180 °C for 120 s had the most stable gluten and protein secondary structure and the lowest fat content [7]. The common mechanism of oil absorption is illustrated in Figure 1. During deep-frying, the rapid evaporation of water on the surface of battered food results in the hardening of the surface and formation of a porous structure, and oil penetrates the food through pores. Moreover, high temperatures promote hydrolysis, polymerization, and oxidation of fats and oils, and the decomposition of triglycerides into polar mixtures (e.g., bis-triglycerides, mono-triglycerides, free fatty acids, and glycerol) promotes the formation of surfactants, accelerates oil degradation, and reduces interfacial tension between oils and foods [7,8]. These effects result in the attachment of large amounts of fats and oils to the surfaces of deep-fried pasty food products. In addition, water vapor condenses during the cooling phase, lowering pressure inside pores and creating a “vacuum effect,” where low positive vapor pressure inside pores forces frying oil through the pores and into the food. The presence of these mechanisms suggests that a dense shell prevents water migration and fat absorption. In recent years, the addition of functional ingredients (e.g., hydrophilic colloidal polysaccharides and proteins) to batter has become a major focus of research on methods for reducing fat absorption in deep-fried battered foods by changing pore size in shells [9]. Combinations of hydrocolloids and starch can exert more considerable synergistic effects than single-component systems, filling in starch granule fragments and resulting in a reduction in voids in the starch gel and the formation of a tight gel network structure through hydrogen bonding and van der Waals forces [10,11]. These synergistic effects enhance the viscosity, rheological behavior, gel strength, and thermal stability of starch-based materials [12]. Cui et al. [8] found that the addition of 0.2% of xanthan gum to the outer breading batter of deep-fried fish pieces as an oil barrier-forming ingredient of deep-fried fish pieces enhanced the viscosity of the batter and improved the rheological behavior of the batter improved the shell stability, and the oil penetration of deep-fried fish pieces was effectively inhibited.

Cassia gum (CG) is a neutral polysaccharide extracted and isolated from the endosperm of cassia seeds in the legume family [13]. Its main structure is composed of galactomannan, which consists of a β-(1-4)-D-mannan backbone and a single α-(1-6)-linked D-galactose side chain, and has a lower galactose content than other commercial galactomannans [14]. The abundant galactose side chains hinder synergistic gelation effects with anionic polymers, thus amplifying the pronounced impact of CG in synergistic interactions [4]. Studies have reported that CG readily swells in water and achieves functionalities such as thickening, freeze–thaw stability, synergistic gelatinization, and film formation through gelation [11]. Galkowska [15] investigated the effects of different concentrations of CG on the gelatinization, rheology, and structural properties of potato and corn starch; they found that CG and starch had a strong synergistic effect, which improved the viscosity, rheology, and gelatinization of starch. Cao et al. [16] made an edible oil packaging film from cassia bean gum, which has a higher barrier property and heat-sealing properties. To date, research on CG mainly focuses on its extraction and purification and its physiological activity, and the application of CG to fried food has not been investigated.

The aim of this experiment was to investigate the effects of different concentrations of CG (0%, 0.2%, 0.4%, 0.6%, 0.8%, and 1%) on the rheological behavior and structural properties of batter. The thermal stability, moisture state, microstructure, and lipid penetration of shells after frying were analyzed, and the lipid penetration mechanism was explored. The findings are intended to provide a scientific basis for the production of low-fat fried small yellow croaker.

## 2. Results and Discussion

### 2.1. Batter Pick-Up and Viscosity

The batter pick-up and batter viscosity show a positive correlation; that is, the higher the batter viscosity, the more batter is attached to the food, and the batter pick-up correspondingly increases [17]. The results of the effect of CG on batter viscosity and batter pick-up are shown in Table 1. The viscosity of the batter increases with the increase in CG addition. This may be because the long-chain molecules in CG, after dissolving in water, combine with starch to form a three-dimensional network structure through hydrogen bonds and intermolecular forces. The higher the concentration, the denser the cross-linking between molecules, resulting in an increase in the flow resistance of the fluid, which is macroscopically manifested as an increase in viscosity. The batter adhering to the small yellow croaker increases, leading to an increase in batter pick-up.

### 2.2. Dynamic Rheological Analysis

**Figure 2:**
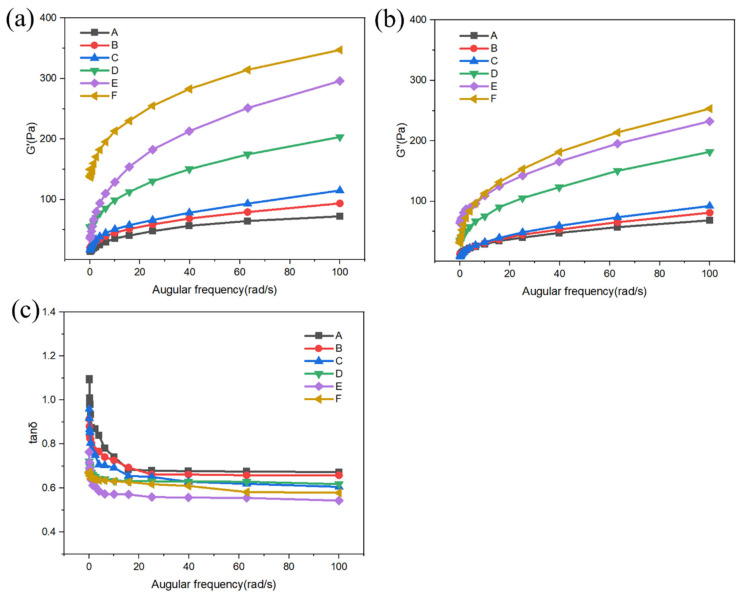
Rheological properties of batters, G′ (**a**), G″ (**b**), and tanδ (**c**). A–F denote 0%, 0.2%, 0.4%, 0.6%, 0.8%, and 1% CG, respectively.

Dynamic rheological properties determine the mechanical behavior of a system under alternating stress (or strain) and can be used to determine the viscoelasticity of different samples [18]. The effect of CG on the dynamic rheology of the batter is illustrated in Figure 2. The G′ and G″ values of the batter increased with CG concentration, indicating that CG contributed to the stability of the batter under external forces. This effect may be due to the fact that the starch molecules and CG were bonded through hydrogen bonding, which led to an increase in the number of entanglement sites in the starch–CG system and the formation of a more stable three-dimensional mesh structure with enhanced gelling properties. These effects enhanced the viscoelasticity of the batter. Meanwhile, the G′ value of this batter was always higher than the G″ value; that is, the δ was less than 1, indicating that the batter exhibited a firm elastic behavior [19]. Compared with the batter without CG, the batter with CG showed a considerable decrease in δ value, indicating that CG effectively promoted the formation of a composite gel, which formed a complex with proteins and starch in the batter and increased the polymer content and degree of polymerization [20]. These effects led to the formation of a stable gluten network structure in the batter.

### 2.3. X-Ray Diffraction

**Figure 3:**
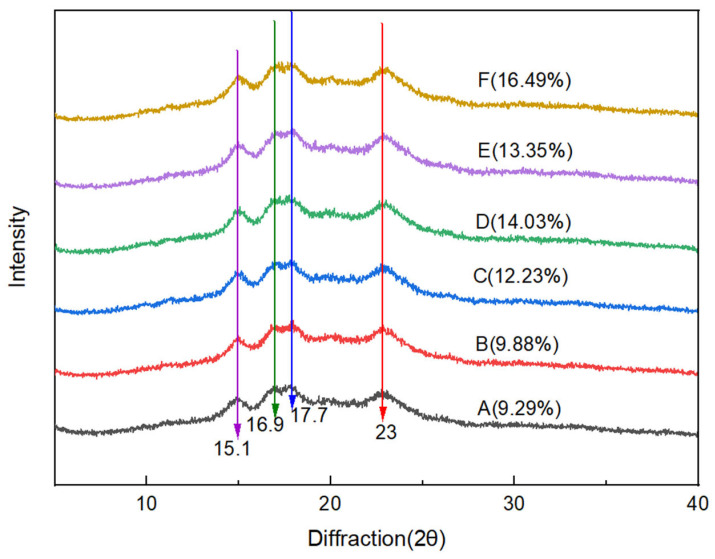
XRD pattern of the batter. A–F indicates 0%, 0.2%, 0.4%, 0.6%, 0.8%, and 1% CG, respectively.

As shown in Figure 3, all the batter samples exhibited similar features: strong diffraction peaks were observed at 15.1° and 23°, and the main diffraction double peaks were observed at 16.9° and 17.7°. These results indicate that the batter samples belonged to a typical A-type crystal structure [21]. Although the addition of CG changed the peak areas, the crystalline type did not change. The RC values (9.29–16.49%) of the batter samples increased with CG concentration, indicating that the addition of CG promoted the binding and rearrangement of starch molecules and contributed to the ordered double-helix and internal three-dimensional network structures [22]. These findings are in agreement with the observation of the samples’ microstructure in Section 2.9, confirming the enhancement of interaction forces (hydrogen bonding and hydrophobic interactions) between the starch gel systems after the addition of CG [19].

### 2.4. FTIR

**Figure 4:**
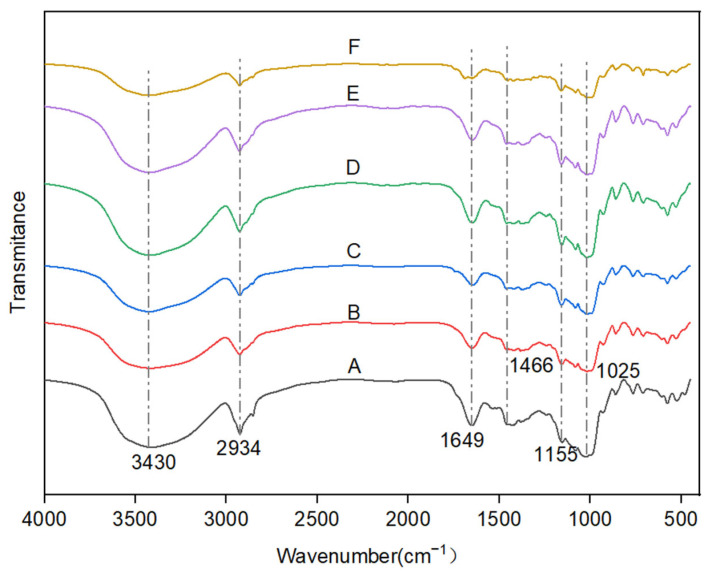
FTIR spectra of the batters. A–F indicate 0%, 0.2%, 0.4%, 0.6%, 0.8%, and 1% CG, respectively.

FTIR spectroscopy is commonly used to study the functional groups of starch [23]. As shown in Figure 4, no new peaks appeared in the IR spectra of all batter samples with CG (0.2–1% CG) and those without (0% CG), implying that the interaction of CG with starch was predominantly noncovalent (including hydrogen bonding, hydrophobic interactions, and other forces) [24]. In addition, no new covalent bonds were observed. The absorption peak at 3430 cm^−1^ was related to the O-H stretching vibration, and the peak shape of the absorption band broadened with increasing CG concentration, possibly because the hydroxyl group of CG formed hydrogen bonds with starch molecules. These bonds increased the vibration of the O-H group and enhanced the intensity of the absorption peak [25]. One sample showed a peak at 2934 cm^−1^, which was attributed to the stretching vibration of C-H. The absorption peak at 1649 cm^−1^ was related to the water adsorption in the amorphous region of the starch molecule, and the wavelength peak at 1649 cm^−1^ was blue-shifted after the addition of CG. This shift indicated that the addition of CG improved the water retention capacity of the system. The peak at 1466 cm^−1^ may be related to the bending vibration of C-H, and the absorption peak was widened by the addition of CG. The peaks at 1155 and 1025 cm^−1^ were attributed to C-H bending and C-O stretching vibrations.

### 2.5. DSC

**Figure 5:**
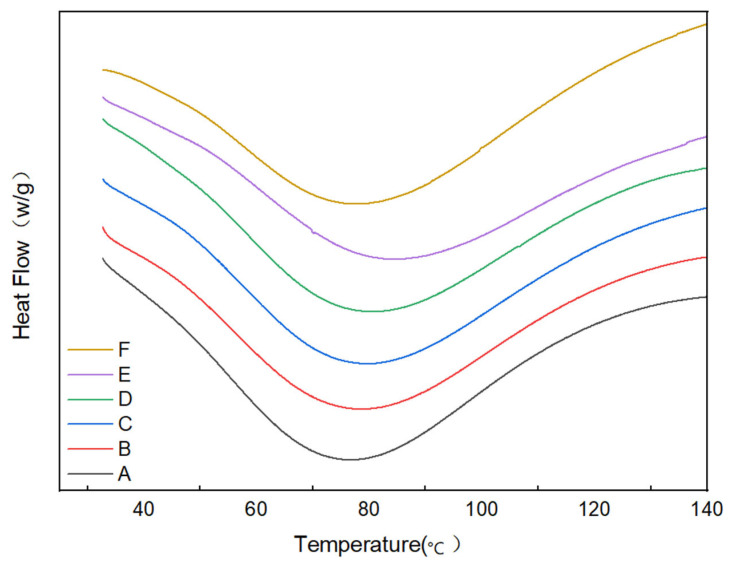
DSC thermal analysis of the batter. A–F indicates 0%, 0.2%, 0.4%, 0.6%, 0.8%, and 1% CG, respectively.

The DSC heat flow curves of the batters are shown in Figure 5, and the thermal transition parameters are shown in Table 2. The ΔH value is linked to the crystallinity of starch, and a low ΔH value reflects the loss of the double helix in a starch molecule and the disruption of crystalline regions [25]. With regard to the batters, a low ΔH value indicated susceptibility to gelatinization. As indicated in Table 2, the Tp and ΔH of the batter increased considerably with increasing CG concentration in a range of 0–0.8%. This increase may be attributed to the binding of CG to starch through hydrogen bonding and intermolecular forces. The complex enhanced intermolecular association and produced gelation, prolonging batterization. Meanwhile, CG promoted the formation of a branched starch double-helix structure, which increased the proportion of crystalline regions and enhanced thermal stability [26]. This observation is consistent with the elevated XRD crystallinity in Section 2.3. However, the Tp and ΔH of the batter decreased instead when CG concentration was increased to 1%. This effect may be due to the formation of an extremely compact gel network at considerably high GC concentrations, which limited the swelling of starch granules, collapse of the structure upon heating, and release of stored energy.

### 2.6. TGA

**Figure 6:**
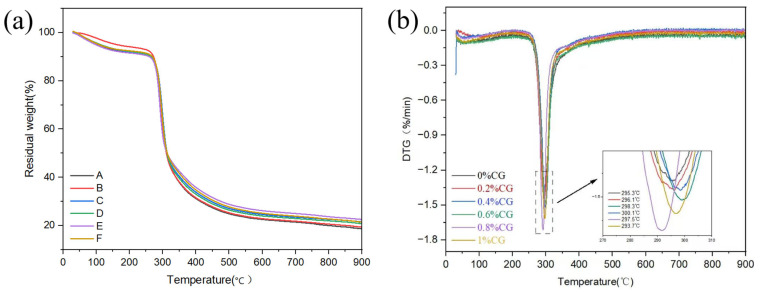
TGA (**a**) and DTG curves (**b**) of the shells. A–F denote 0%, 0.2%, 0.4%, 0.6%, 0.8%, and 1% CG, respectively.

The thermal stability of the samples can be assessed by measuring weight loss at increasing temperatures [27]. Figure 6 shows the TGA results and corresponding TGA curves of the samples in the first stage when the temperature is in the range of 50–250 °C. The weight loss of the samples can be attributed to the evaporation of water [28]. In the second stage, when the temperature was in a range of 250–400 °C, the weight loss was mainly related to the thermal degradation of the important components of WF (starch and gluten proteins). At this stage, the shells without CG (0% CG) exhibited considerable mass loss compared with the other CG-containing shells, indicating that CG improved the thermal stability of the shells. This improvement was mainly attributed to the interaction between CG and starch, which formed a dense gel structure that improved the thermal stability of the complex. The weight loss of the different samples was relatively constant in a range of 400–550 °C (final stage) during the third stage. The shell samples containing CG were heavier than those without CG, indicating that the shells were less likely to be decomposed by the addition of CG, and the amount of residue left after the thermal decomposition of the samples increased [29]. The DTG curve represents the relationship between the rate of weight change and temperature [30]. As shown in Figure 6b, the temperature of the maximum degradation rate of the shells exhibited an increase followed by a decrease as the CG concentration increased, and the temperature corresponding to the maximum degradation rate of the samples reached a maximum of 300.1 °C when CG concentration was 0.6% because CG at low concentrations binds to starch molecules and forms a dense gel structure. This structure enhances the thermal stability of the shell and complicates thermal decomposition, increasing the temperature of the maximum degradation rate [31]. Excessively high CG concentrations may lead to the localized disruption of the gel network, increasing susceptibility to decomposition and thus lowering the temperature of the maximum degradation rate. These findings suggest that CG improves the thermal stability of shells.

### 2.7. Moisture Content and Fat Content

**Figure 7:**
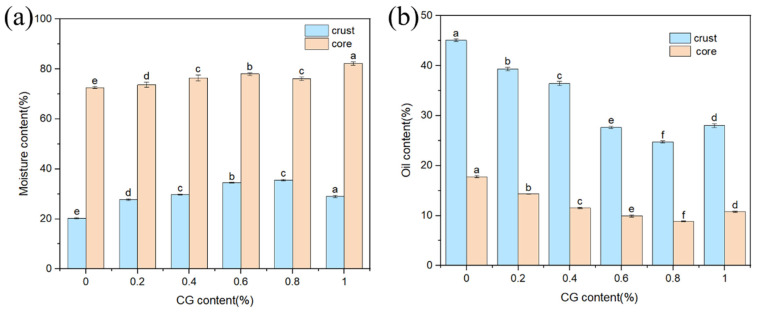
Moisture content (**a**) and fat content (**b**) of BFYC shell and fish flesh. Different letters indicate significant differences, *p* < 0.05.

As shown in Figure 7a, the water content of the shell first increased and then decreased with increasing CG concentration. This behavior may be attributed to the formation of a dense layer composed of CG and starch at low temperatures. The layer retarded the evaporation of water and increased the water content of the shell [32,33]. When an extremely high concentration of CG was added, the excess colloid led to the phase separation of the starch–colloid system (the colloid and starch competed for water and space), the structure of the composite network was disrupted, and the water-holding capacity decreased, resulting in water loss. The moisture content of the internal fish meat showed a progressive increase with increasing CG concentration owing to the formation of a viscoelastic gel layer of CG that covered the surface of the fish meat during deep-frying. Additionally, the layer hindered the migration of internal water to the outer shell, reducing the infiltration of external oils and fats.

From Figure 7b, it can be seen that the fat content of both shell and fish meat showed a tendency to decrease and then increase with the increase in CG addition. In this case, the fat content of the shell decreased from 45.07% to 24.75%. This was due to the fact that when the CG addition was low (in the range of 0–0.8%), CG was able to form a gel rapidly under high-temperature conditions, which strengthened the tight intermolecular bonding in the batter and formed a dense network structure. This structure significantly enhanced the barrier function of the shell and reduced the voids left behind after water loss during frying [34], thus effectively inhibiting the penetration of oil into the shell and fish interior by capillary action. However, the fat content of the shell instead increased to 28% as the CG addition increased to 1%. This may be due to the fact that CG, as a hydrophilic colloid, has an excessive ability to bind water, leading to its binding of excessive water. It played an obvious inhibitory effect on the swelling and pasting of starch granules, hindered the formation of the gel layer, and led to the emergence of clefts or localized loose areas in the gel network [35], which formed microporous channels and provided pathways for the penetration of fats and oils, thus increasing the fat content in the deep-frying process.

### 2.8. Moisture Status

**Figure 8:**
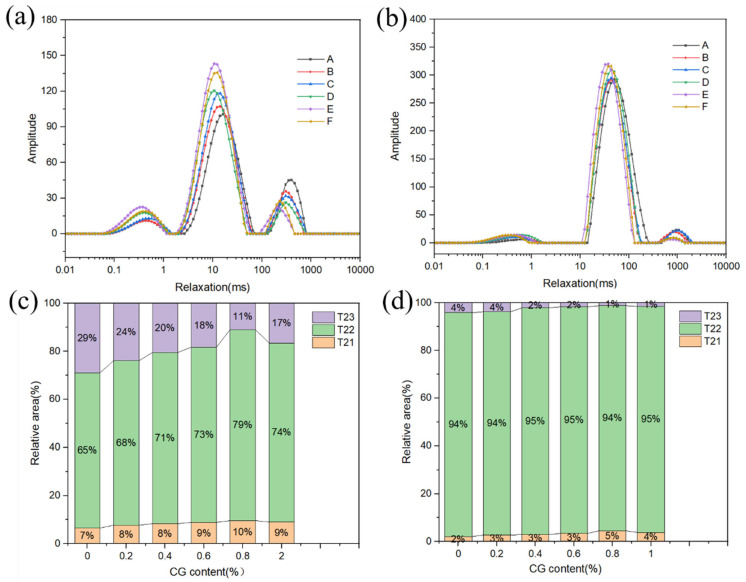
Inversion spectra of transverse relaxation time (T2). (**a**,**b**) Moisture states of BFYC shell and fish flesh, respectively, and (**c**,**d**) relative peak area ratios of T21, T22, and T23 for BFYC shell and fish flesh, respectively. A–F denote 0%, 0.2%, 0.4%, 0.6%, 0.8%, and 1% CG, respectively.

The transverse relaxation time (T2) represents the degree of freedom of water. The shorter the T2, the lower the degree of freedom of water, and the tighter the combination of water with the food components [36]. Figure 8a,b shows the moisture conditions of the BFYC shell and fish meat, while Figure 8c,d shows the relative peak area ratios of T21, T22, and T23 in the BFYC shell and fish meat. T21 (0.01–1 ms) represents deeply bound water, which is mainly water tightly bound to starch and proteins. T22 (1–10 ms) represents weakly bound water, which is a part of water with a degree of freedom intermediate between the strongly bound water and free water and is mainly water-bound to the multimolecular layer between large molecules, such as starch and protein. T23 (100–1000 ms) is free water, which is more mobile [37].

Figure 8a,b shows that the time of appearance of the T21, T22, and T23 peaks of BFYC shells and fish meat shifted to the left as CG concentration increased, indicating that CG adsorbed water molecules through hydrogen bonding and intermolecular forces, enhanced the ability of amylose gel to bind water, and reduced the mobility of water [38], resulting in the shortening of T2 relaxation time. When the CG concentration was over 1%, the peak of T23 shifted to the right, possibly because of the excessive hydrophilic colloids competing with starch and gluten for binding water molecules. In addition, excessive colloid concentration was found in local areas, triggering phase separation (e.g., colloid aggregation or starch–colloid phase separation), which disrupted the ordered structure of the starch gel and increased the degree of water freedom [39]. Figure 8c shows that most of the water in the shell was in the form of weakly bound water, which accounted for more than 70% of the peak area ratio. As CG concentration further increased, a dynamic transformation between weakly bound water and free water was observed in the shell [40]. The proportion of weakly bound water increased, whereas the proportion of free water decreased. This phenomenon may be attributed to the hydrophilic nature of CG, whose molecular chains are rich in hydroxyl and other polar groups, which can be bound to water molecules through hydrogen bonding and van der Waals forces. Moreover, colloidal molecules adsorbed free water (T23), which originally existed in the form of large pores or interfaces, and convert it into weakly bound water (T22) bound by the colloidal network. Therefore, the presence of CG enhanced the water-holding capacity of the starch gel system. The highest moisture content of the shells was observed after 0.8% CG was added, indicating that the addition of CG mitigated water loss in BFYC during frying and effectively inhibited the penetration of oil and grease. This finding is consistent with the measurements of oil and moisture content in Section 2.7.

### 2.9. Scanning Electron Microscopy (SEM)

**Figure 9:**
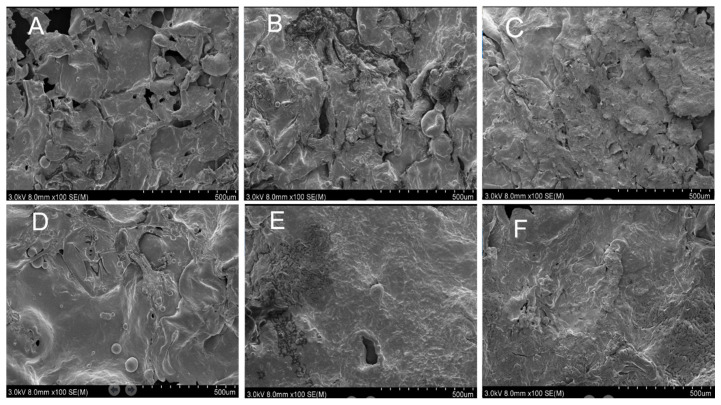
Scanning electron microscopy (SEM) images of BFYC shells. (**A**–**F**) denotes 0%, 0.2%, 0.4%, 0.6%, 0.8%, and 1% CG additions, respectively. The SEM images were taken at 100× magnification.

The microstructural features of BFYC can intuitively reflect its surface pore distribution and roughness changes. The surface morphology of the samples with different CG concentrations (Figure 9) was observed by scanning electron microscopy (SEM). The shell surface gradually tended to be smooth, and the number of pores decreased considerably with increasing CG concentration. The mechanism of pore formation on the surface of the samples was mainly attributed to the formation of pores as a direct result of the dramatic evaporation of surface water during frying [41,42]. These pores provided channels for the penetration of frying oil. However, after the addition of CG, the mannose backbone in CG combined with starch molecules through hydrogen bonding and van der Waals forces, forming a continuous and dense gelatinous membrane [43]. This membrane structure effectively blocked the penetration of oil. In addition, CG considerably enhanced the water-holding capacity of the batter and reduced the evaporative loss of water during frying, inhibiting the formation of pores [43,44]. Therefore, the moderate addition of CG can considerably reduce the porosity of BFYC shells, which, in turn, reduces the intake of fats and oils.

### 2.10. Grease Penetration

**Figure 10:**
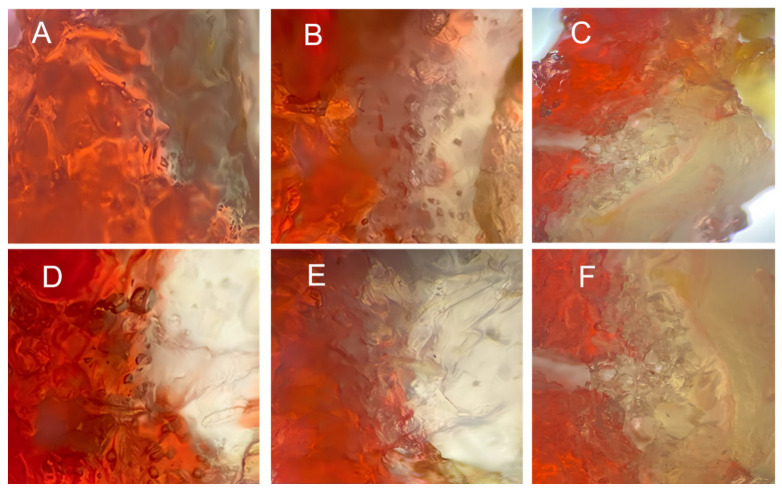
Sudan Red staining of BFYC. (**A**–**F**) indicates 0%, 0.2%, 0.4%, 0.6%, 0.8%, and 1% CG, respectively. Optical microscopy images were taken in reflection mode at 4× magnification.

Sudan Red B dye is thermally stable and fat-soluble and has a permeation behavior similar to that of frying oil. It is commonly used to reflect the distribution and permeation of fats and oils in fried foods [45]. The BFYC cross-section staining observed by light microscopy is shown in Figure 10. A higher degree of dye penetration was observed in the control group owing to the loose structure of the starch gel formed by the batter composed of WF and TS, which facilitated water evaporation and the formation of large pores. During the cooling process, higher amounts of fats and oils penetrated the fish pieces because of negative pressure. As CG concentration increased, the area of the Sudan Red-stained area gradually decreased in size, and the oil was mainly distributed in the shell and the junction between the shell and meat rather than inside the fish. This finding indicated that the addition of CG exerted an inhibitory effect on oil penetration in BFYC. The pores in the structure of the shell’s surface were reduced, and the density of the structure increased. Therefore, the amount of oil that penetrated the fish meat was reduced. These findings are in line with the results demonstrating the effect of CG addition on the oil content of BFYC.

## 3. Conclusions

CG improved the viscoelasticity of the batter by combining with starch through noncovalent interactions, changing the structural properties of the starch gel, forming a dense three-dimensional network structure, and increasing the percentage of deeply bound water and weakly bound water in the batter. It also enhanced the water-holding capacity of the system, reducing the loss of water during deep-frying, improving the thermal stability of the batter, slowing down the pasting process, and inhibiting the formation of pores through water evaporation. Hence, penetration by oil and grease was mitigated. SEM and Sudan Red-staining experiments confirmed that 0.8% CG addition resulted in the lowest shell porosity, which represented a considerable reduction in the penetration depth of oil and grease and a reduction in shell fat content of approximately 40% compared with the control group. This study provides a theoretical basis for the development of low-fat fried foods. The synergistic effect of CG with other hydrophilic colloids and its universality in different fried food systems can be further explored in the future to promote its application in industrialized production.

## 4. Materials and Methods

### 4.1. Materials

Soybean oil was purchased from Yihai Kerry Foods Company Limited (Shanghai, China); wheat flour (WF) and ta0pioca starch (TS) were purchased from Great Southwest Flour Co. (Rizhao, China); Sudan B was purchased from Tianjin Zhiyuan Chemical Reagent Company Limited (Tianjin, China); petroleum ether and potassium bromide were purchased from Guangdong Qianjin Chemical Reagent Company Limited (Guangzhou, China). All the chemicals and reagents used in this study were of analytical grade, and all aqueous solutions were prepared using deionized water.

### 4.2. Batter Preparation

The method described by Cui [8] was adopted with slight modifications. A mixture was prepared by combining WF (50 g), TS (38 g), salt (1 g), and deionized water (100 g). CG was added at varying concentrations to the mixture (0%, 0.2%, 0.4%, 0.6%, 0.8%, and 1% in terms of total batter weight). Each mixture was homogenized using a thermostatic magnetic stirrer (R-30, Yuhua Instrument Co., Ltd., Shanghai, China) at 1500 rpm for 10 min at 25 °C and covered with plastic wrap to prevent water evaporation.

### 4.3. Preparation of Fried Battered Small Yellow Croaker (BFYC)

The method described by Feng [35] was adopted with slight modification. Thawed small yellow croaker was immersed in the prepared batter for 10 s and then drained for 15 s. Dipping was repeated until the batter was evenly coated on the surface of the fish pieces, which were deep-fried in a deep fryer (Jiangmen Aokoding Equipment Co., Ltd., Guangzhou, China) at an oil temperature of 160 °C for 2 min and then at 190 °C for 30 s. The fish samples were removed from the oil and allowed to cool at room temperature for 1 h to facilitate the removal of excess surface oil. Indexes were determined during the later stages of deep frying.

### 4.4. Batter Pick-Up

The method described by Salvador [46] was adopted with slight modification. The small yellow croaker was immersed in the batter for 10 s, removed, and allowed to drip naturally for 10 s. The pick-up was used to indicate the amount of batter that adhered to the small yellow croaker and was calculated using the following equation:

(1) $$\mathrm{Pick}\text{-}\mathrm{up}/\%=\frac{m1-m2}{m1}\times 10$$

where m1 and m2 represent the weight of BFYCs (g) and small yellow croaker (g), respectively.

### 4.5. Viscosity

The method described by Sun [47] was adopted with slight modification. The batter was stirred thoroughly at room temperature with a viscometer (NDJ-8S, Shanghai Analytical Bull Leiber Instrument Co., Ltd., Shanghai, China) and a No. 4 rotor. The height was adjusted until the liquid surface was 1 mm above the rotor. After which, the rotor was operated at 60 r/min for 30 s to ensure a stable reading.

### 4.6. Dynamic Rheological Properties

The dynamic rheological modulus of the batters was determined using the method described by Li [40], and rheological measurements of the batters were carried out at 25 °C with a rheometer (MCR302, Anton Paar Co., Ltd., Graz, Austria). Three drops of batter were added to a plate (40 mm diameter and 1 mm spacing) and covered with a flap. Excess sample material was scraped off, and a ring of mineral oil was applied around the sample to prevent water evaporation. Frequency scanning was performed under the following conditions: temperature, 25 °C; strain, 0.5%; scanning range, 0.1–100 rad/s. The elastic modulus (G′), viscous modulus (G″), and tangent (tanδ (G″/G′)) data of the samples were recorded.

### 4.7. X-Ray Diffraction Analysis

The method described by Han [18] was adopted with slight modifications. The freeze-dried batter samples were scanned using an X-ray diffractometer (D8 ADVANCE, BRUKER AXS, Karlsruhe, Germany) at a 2θ range of 5–40° at 5°/min. The XRD spectra of the samples were analyzed and recorded using Jade 6.0 and Origin 2021 software. The relative crystallinity (RC) was calculated using the following formula:

(2) $$\mathrm{RC}=\frac{\mathrm{Ac}}{\mathrm{Ac}+\mathrm{Aa}}\times 100$$

where Ac and Aa are the areas of crystalline and amorphous regions, respectively [22].

### 4.8. Fourier Transform Infrared Spectroscopy (FTIR)

The Fourier transform infrared (FTIR) spectra of the samples were recorded using an FTIR spectrometer (Vector 33, Bruker Optics, Ettlingen, Germany) according to the method described by Zou [48]. The lyophilized samples were mixed with KBr in a ratio of 1:100, and the resulting mixture was homogenously ground and pressed into a tablet for measurement. The spectra of the samples were recorded during 32 scans with 4 cm^−1^ resolution and 400–4000 cm^−1^ scanning range.

### 4.9. Differential Scanning Calorimeter (DSC)

The thermal properties of the batters with different CG concentrations were determined using a differential scanning calorimeter (Q200M, TA instrument, New Castle, DE, USA) according to the method described by Zhang [49]. Approximately 3 mg of batter was collected and sealed in an aluminum crucible. Each sample was heated from 25 °C to 140 °C at a rate of 10 °C/min, and the constant flow rate of nitrogen was 50 mL/min.

### 4.10. Thermogravimetric Analysis (TGA)

The method described by Cui [8] was adopted with slight modification. A thermogravimetric analyzer (TGA 8000, Perkin Elmer Instruments, Suzhou, China) was used to determine the thermogravimetric properties of fried battered small yellow croaker (BFYC) shells. The shells were degreased, lyophilized, and pulverized into powder with a high-speed crusher (JYL-C23, Joyoung Co., Ltd., Jinan, China). Approximately 5 mg of the powder was placed in a crucible for analysis, and an empty crucible was used as the control to account for any interference by the crucible itself. Nitrogen was used as the protective gas, and the flow rate was 20 mL/min. The temperature was increased from 20 °C to 700 °C at a rate of 10 °C/min, and then the samples were allowed to cool naturally.

### 4.11. Moisture Content

Approximately 2 g of shell and fish flesh of BFYC were separately placed in an aluminum box and dried in a hot air drying oven (1101-1-BS, Shanghai Yuejin Medical Instrument Factory, Shanghai, China) at 105 °C until a constant weight was reached.

(3) $$Z(\%)=\frac{m1-m2}{m1-m3}\times 100$$

where Z is the moisture content of the shell or fish; m1 is the total mass of the aluminum box and shell or fish before drying (g); m2 is the total mass of the aluminum box and shell or fish after drying (g), and m3 is the mass of the aluminum box (g).

### 4.12. Fat Content

Approximately 2 g of shell and fish flesh of BFYC were wrapped in filter paper separately. The samples were placed in an extraction tube of a Soxhlet extractor (SOX604, Jinan Elva Instrument Co., Ltd., Jinan, China) containing petroleum ether. After 10 h of extraction at 60 °C, the difference in weight between the samples before and after extraction was used to calculate the oil content of the shell and fish flesh.

(4) $$Z(\%)=\frac{m1-m2}{m}\times 100$$

where Z is the fat content of the shell or fish meat; m is the mass of the shell or fish meat (g); m1 is the weight of the extraction bottle (g), and m2 is the mass of the extraction bottle after extraction of fats and oils (g).

### 4.13. Moisture Status Determination

The method described by Zhang [50] was adopted with slight modification. Fried BFYC was separated from the shell and fish meat and chopped, and approximately 2 g of the shell or fish meat was weighed and placed in the probe of a low-field NMR instrument (NMI20-015V-I, Suzhou Neumay Analytical Instruments Co., Ltd., Suzhou, China) for T2 inversion. The diameter was 40 mm. The lateral relaxation time was measured by applying the CPMG sequence, and the magnetic intensity was 0.5 T. The magnetic field was 23.3 MHz. The CPMG sequence was applied to measure the transverse relaxation time at a magnetic intensity of 0.5 T and a magnetic field of 23.3 MHz. Data were recorded and analyzed.

### 4.14. Scanning Electron Microscope (SEM) Observation

The method described by Zhang [51] was adopted with slight modification. The BFYC shell was degreased with petroleum ether and freeze-dried. The powder was adhered to the specimen stage with a conductive adhesive and then sprayed with gold for 90 s. The microstructural features of the shells were then observed at 100× magnification under a 7.0 KV hot field scanning electron microscope (EM-30 Plus, Korea COXEM Corporation, Daejeon, Republic of Korea).

### 4.15. Sudan Red Staining

Sudan Red B weighing 1.75 g was added to 3.5 L of soybean oil and heated at 60 °C for 6 h to completely dissolve, and the samples were prepared by frying with dyeing oil according to the production process in Section 2.3. The prepared samples were cut into 2 mm-thick slices, stained, and observed under an optical microscope with a 4× objective [52].

### 4.16. Statistical Analysis

All the test samples and indicators had three replicates. Correlation results were expressed as mean ± standard deviation. Data were analyzed for statistical significance using one-way ANOVA (*p* < 0.05). Significant differences between values were analyzed and compared using IBM SPSS Statistics 26. All data were obtained using Origin 2021.

## Figures and Tables

**Table 1:** Effect of cassia gum (CG) addition on batter viscosity and paste-holding rate.

CG Content (%)	Viscosity (cP)	Batter Pick-Up (%)
0	2131.45 ± 45.68 ^f^	38.68 ± 0.46 ^e^
0.2	3224.93 ± 94.73 ^e^	39.85 ± 0.59 ^e^
0.4	5150.50 ± 137.46 ^d^	42.89 ± 0.62 ^d^
0.6	8103.49 ± 19.55 ^c^	52.35 ± 0.53 ^c^
0.8	11,882.19 ± 171.38 ^b^	70.48 ± 1.18 ^b^
1	15,567.00 ± 166.47 ^a^	84.81 ± 1.61 ^a^

Note: Different letters in the upper right corner of the numbers in the table indicate significant differences (*p* < 0.05).

**Table 2:** Denaturation temperature (Tp) and enthalpy (ΔH) values of DSC.

CG Content (%)	T_p_ (°C)	ΔH (J/g)
0	78.23 ± 0.84 ^c^	17.329 ± 0.12 ^e^
0.2	78.93 ± 0.34 ^bc^	17.97 ± 0.17 ^e^
0.4	80.84 ± 1.05 ^abc^	19.96 ± 0.35 ^d^
0.6	81.81 ± 1.24 ^ab^	21.14 ± 0.29 ^c^
0.8	82.08 ± 1.09 ^a^	23.35 ± 0.25 ^a^
1	80.31 ± 0.34 ^abc^	22.15 ± 0.30 ^b^

Note: Different letters in the upper right corner of the numbers in the table indicate significant differences (*p* < 0.05).

## Data Availability

The original contributions presented in this study are included in the article. Further inquiries can be directed to the corresponding author.
